# Management of Lobular Capillary Hemangioma Using Diode Laser: A Case Report

**DOI:** 10.7759/cureus.60068

**Published:** 2024-05-10

**Authors:** Keerthika Varadhan, Ramnath Elangovan, Selvakumar J, Kowsalya Nallathambi, Dinesh C Maganti, Dona Soman

**Affiliations:** 1 Department of Periodontics, Adhiparasakthi Dental College and Hospital, Melmaruvathur, IND; 2 Department of Periodontology, School of Dentistry, University of Rwanda, Kigali, RWA; 3 Department of Periodontology and Community Dentistry, School of Dentistry, University of Rwanda, Kigali, RWA; 4 Department of Prosthetic and Restorative Dentistry, School of Dentistry, University of Rwanda, Kigali, RWA

**Keywords:** oral lesion, benign vascular tumor, diode laser, dental laser, lobular capillary hemangioma

## Abstract

This case report focuses on the clinical development of a 32-year-old female patient's lobular capillary hemangioma and provides valuable insights into the atypical nature of this tumor. Low-level laser therapy (LLLT) that follows diode laser intervention can be regarded as a novel and evidence-based approach to therapeutic management. The application of a diode laser causes the vascular elements that comprise the bulk of the lesion to coagulate, which in turn causes the lesion's size to decrease. The biological processes that lead to quick tissue regeneration are also activated by LLLT. The suggested therapeutic approach ensures that the patient will heal in the best possible way while also optimizing their comfort and safety. It extends beyond the mere removal of wounds. The case report demonstrates how well dual laser therapy works to lessen common postoperative issues that are commonly seen in traditional surgical therapies for lobular capillary hemangioma such as excessive bleeding and infection. The precise application of the diode laser minimizes damage to surrounding tissues, thereby enhancing the healing process. Additionally, following surgery, LLLT helps reduce pain and inflammation, which improves patient outcomes. The potential of diode laser and LLLT therapies for treating vascular lesions, including lobular capillary hemangioma, is evidenced by their therapeutic advantages. This encourages wider clinical applications and field research. The presented case report offers valuable clinical significance by highlighting an innovative therapeutic approach for lobular capillary hemangioma, a vascular lesion that can present challenges in management.

## Introduction

Lobular capillary hemangiomas (LCHs) are noncancerous growths of blood vessels that frequently occur in the skin and mucous membranes. They seem to occur more often in the head and neck area, particularly in the oral cavity [1]. According to the existing literature, there have been no reports of LCH originating from the mandible. The infrequency of this condition, combined with the lesion's ability to imitate more ominous entities, such as cancer, might be a challenge for the clinician in terms of treatment decisions. Similar to other reactive or hyperplastic growths, LCH is believed to occur due to an excessive formation of new blood vessels in response to various stimuli, such as infection, local irritation or injury, and hormonal activity. The latter is likely the reason why LCH tends to occur more frequently in the head and neck regions [2]. LCH was previously mislabeled as a "pyogenic granuloma," which is an incorrect term for this harmless condition [3]. LCH is most frequently found in the gingiva, with extra-gingival cases being uncommon. The current example is unequivocally located outside the gum tissue, namely, on the lip. This example highlights the importance of including LCH as part of the clinical and histological differential diagnosis when assessing vascular lesions.

## Case presentation

**Figure 1:**
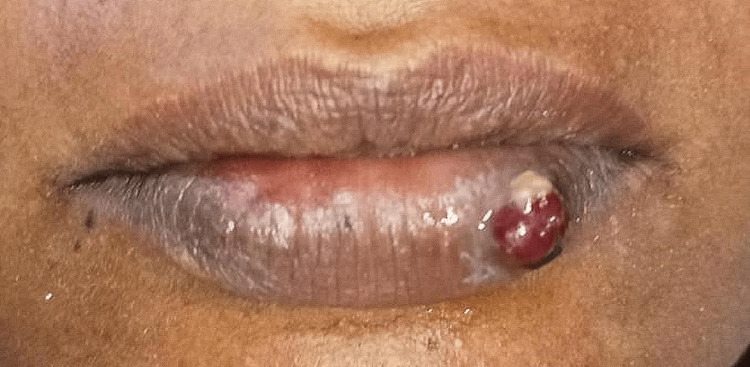
Preoperative image of the lesion in the lower left lip region.

A 32-year-old female patient presented to the Department of Periodontics with a chief complaint of edema in her lower lip region, persisting for the last three weeks. She provided a detailed account of a protrusion on the upper lip, expressing her primary concern about its appearance. The patient has no pertinent medical background. A dental procedure, including scaling, was performed two years ago. The patient does not report any other instances of lip-biting or injuries. No notable discoveries were made during the inspection of the external parts of the body. The submandibular lymph nodes were detectable through touch. Upon examination, the lesion appeared as a single, stalk-like growth with a round form. It had a reddish-pink color, a well-defined edge, and an uneven surface. The dimensions of the lesion in the lower left lip region were approximately 1 × 1 cm (Figure 1).

**Figure 2:**
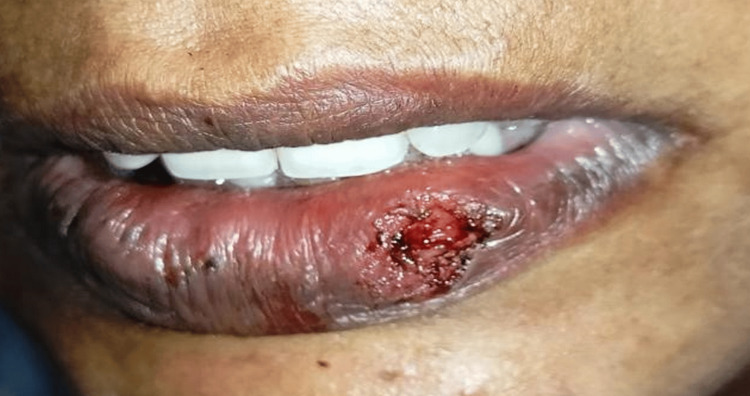
After excision of the lesion with a diode laser (Indilase 980 nm).

All the results identified during the inspection were verified through palpation. There was an absence of discharge; it had a smooth texture and did not cause any pain when touched. Consequently, a temporary diagnosis of mucocele was established, and a surgical removal procedure with a diode laser was scheduled. A surgical procedure known as an excisional biopsy was performed following the application of local anesthesia, namely, a solution containing 2% lignocaine and a concentration of 1:80000 epinephrine. The excision of the lesion was performed using a diode laser called Indilase 980 nm, operating at a power of 1 W in gated pulse mode. This is illustrated in Figure 2.

**Figure 3:**
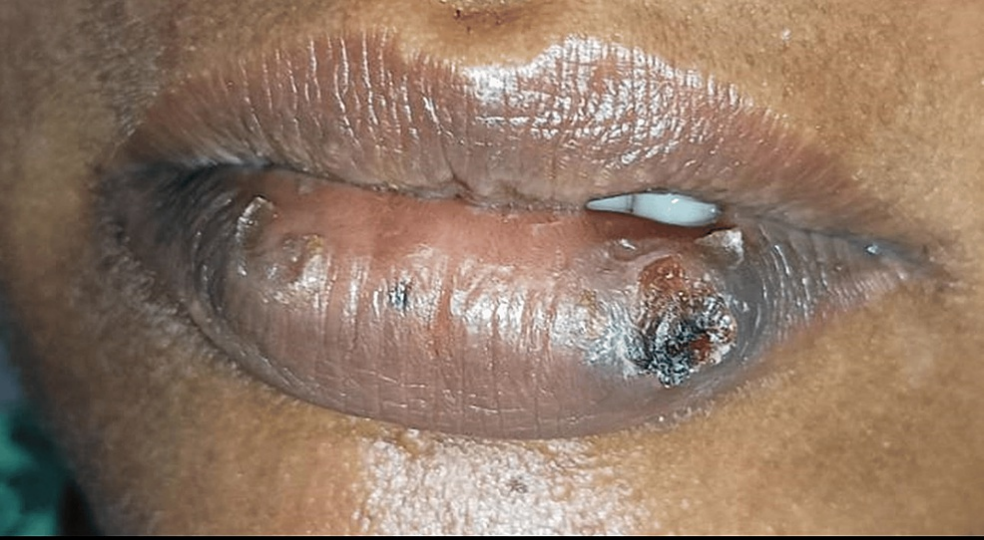
Postoperative image of excision site after 10 days.

**Figure 4:**
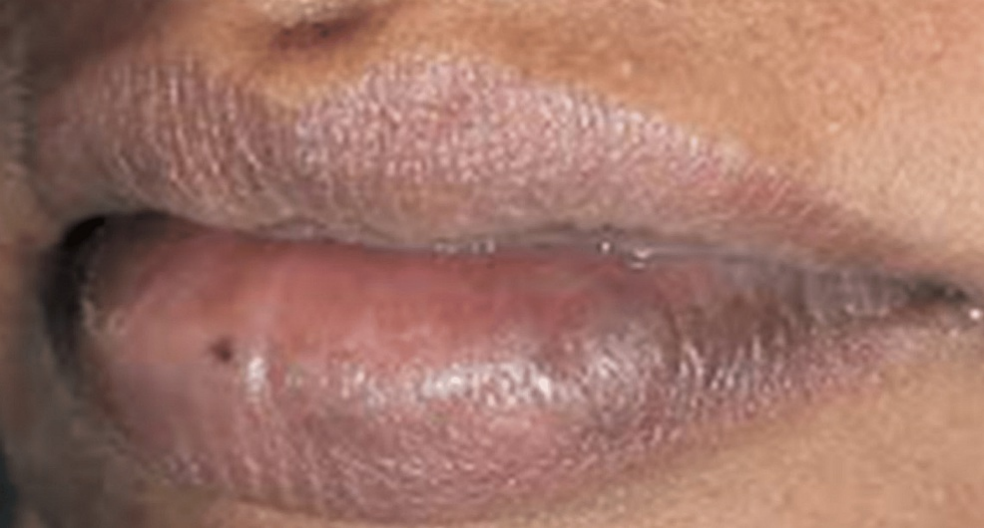
Review after six months of follow-up.

Following the removal of the tissue, there was a significant amount of bleeding. Subsequently, the bleeding was stopped, and the removed region was treated with low-level laser therapy (LLLT) using a configuration of 780 nm, 60 mW, and 3.0 J/cm^2^.The excised tissue was immersed in a 10% formalin solution and dispatched for histological analysis. The patient was comfortable; there was no hemorrhaging, and no stitches were necessary. The patient was instructed to use ibuprofen 400 mg thrice daily without any prescription for antibiotics. The patient was summoned for a follow-up examination after 10 days and again after six months. The healing process was successful, with no postoperative problems or recurrence observed after a period of six months (Figure 3 and Figure 4).

**Figure 5:**
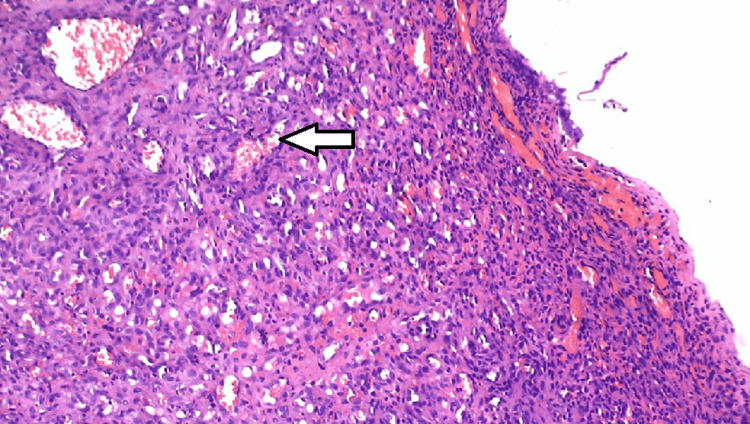
Histopathological image. The arrow indicates areas where chronic inflammatory cells like lymphocytes are scattered, and acute inflammatory cells are present in the ulcerated area.

The H&E-stained slices of the provided soft tissue specimen showed an orthokeratinized and parakeratinized stratified squamous epithelium that is ulcerated in one region. The epithelium surface seems to have uneven folds and is uniformly atrophied in many regions while being hyperplastic in some focused locations. The underlying connective tissue appears to be fragile, uniform, composed of cells, and containing blood vessels. The entire connective tissue exhibited a dispersion of capillaries of all sizes, ranging from small to large. These capillaries were accompanied by extravasated red blood cells and a plentiful presence of proliferating cells around the capillary gaps. These proliferating cells resembled endothelial cells and displayed scattered mitotic patterns. Figure 5 shows the presence of chronic inflammatory cells, such as lymphocytes, dispersed across the area. In addition, the ulcerated area contains predominantly acute inflammatory cells, specifically neutrophils.

## Discussion

Hemangiomas constitute 7% of the total number of benign tumors that occur during infancy and childhood. Moreover, while hemangiomas occurring in the head and neck region are quite prevalent, constituting at least one-third of all hemangiomas in humans [4], they can be easily mistaken for other conditions due to their ability to histologically resemble other abnormalities [5]. Vascular malformations refer to localized or widespread abnormalities that occur during the development of an embryo. Based on their clinical and histological characteristics, they can be categorized as capillary, lymphatic, venous, arterial, or a mix of these types [6]. In the current scenario, pyogenic granuloma (LCH) is a type of vascular lesion that exhibits excessive growth. It is frequently misdiagnosed as hemangioma due to its similar histological characteristics, both being classified as capillary hemangioma. Pyogenic lesions frequently exhibit a diminutive, pedunculated morphology. This lesion is commonly referred to by pathologists as capillary hemangioma, granuloma type, or LCH [7].

This case report highlights the significance of utilizing minimally invasive methods in dermatology by demonstrating the effective use of diode laser therapy in managing LCH. Diode laser therapy provides numerous benefits compared to conventional surgical removal, such as accurate focus on vascular tissue, minimal harm to adjacent healthy skin, and a decreased likelihood of scarring [2]. Diode laser treatment utilizes the photothermal properties of laser light to cause coagulation in the blood vessels of a hemangioma. This process effectively reduces and resolves the size of the lesion. This method guarantees both excellent aesthetic results and reduces patient discomfort while also speeding up the recuperation process after treatment. Furthermore, diode laser therapy can be conducted as an outpatient procedure with the use of local anesthetic, providing convenience and cost-effectiveness for both patients and healthcare professionals.

Moreover, the favorable result of this case report emphasizes the increasing significance of laser therapy in the treatment of diverse dermatological disorders, such as vascular lesions like LCH. With the continuous advancement of technology and the increasing sophistication of laser systems, physicians can utilize these advancements to customize treatment approaches according to the specific needs of each patient. Diode laser therapy is a vital and successful tool for dermatologists to treat patients with vascular lesions. It offers safe and efficient treatment choices due to its versatility and efficacy [5]. Further research and clinical experience will continue to increase our understanding of the best parameters and protocols for diode laser therapy. This will lead to better outcomes and improved patient care in the field of dermatology.

This small lesion is not life-threatening upon presentation and radiograph as it does not indicate any bone involvement. Consequently, emergency surgery to limit hemorrhage is not necessary. As a result, it was determined that the best course of action for this case would be to treat it by a straightforward excision procedure while taking all essential safeguards. The current instance was monitored six months following surgical removal. The healing process was fully accomplished, and no reappearance was detected.

## Conclusions

Dental lasers are effective at surgically removing hematological lesions like LCH from the mouth, as shown in this case report. Photobiomodulation and precision laser excision reduce bleeding risks and speed healing in such cases. The findings inform medical professionals and emphasize the importance of laser-based approaches to improve patient care and outcomes.

The widespread use of dental lasers to eradicate hematological malignancies like LCHs could also change healthcare delivery. These enhancements can improve healthcare efficiency and resource allocation by optimizing processes, minimizing intrusive interventions, and promoting rapid recovery. It is also vital to note that patient satisfaction and treatment worries may improve. Dental professionals can meet the demand for minimally invasive procedures and modern technology by using dental lasers. This meets patients' changing expectations and promotes dentists as innovators in oral healthcare. In addition to the immediate therapeutic benefits shown in this study, laser-based approaches can alter oral surgery and enable a future that prioritizes patient demands and is driven by technology. These dental technology advances can improve treatment efficacy, reduce issues, and raise patients' quality of life.
